# The Relationship Between Concurrent Preoperative Dry Eye Disease and Ascorbic Acid Levels and the Outcomes of Kerato-Lenticule Extraction Surgery

**DOI:** 10.7759/cureus.78430

**Published:** 2025-02-03

**Authors:** Chia-Yi Lee, Shun-Fa Yang, Hung-Chi Chen, Yi-Jen Hsueh, Jing-Yang Huang, Chao Kai Chang

**Affiliations:** 1 Institute of Medicine, Chung Shan Medical University, Taichung, TWN; 2 Department of Ophthalmology, Nobel Eye Institute Taichung Branch, Taichung, TWN; 3 Department of Ophthalmology, Jen-Ai Hospital Dali Branch, Taichung, TWN; 4 Department of Medical Research, Chung Shan Medical University Hospital, Taichung, TWN; 5 Department of Ophthalmology, Chang Gung Memorial Hospital, Linkou, Taoyuan, TWN; 6 Department of Medicine, Chang Gung University College of Medicine, Taoyuan, TWN; 7 Center for Tissue Engineering, Chang Gung Memorial Hospital, Linkou, Taoyuan, TWN; 8 Department of Ophthalmology, Nobel Eye Institute Taipei Branch, Taipei, TWN; 9 Department of Optometry, Yuanpei University of Medical Technology, Hsinchu, TWN

**Keywords:** ascorbic acid, dry eye disease, high myopia, kerato-lenticule extraction, uncorrected distance visual acuity

## Abstract

Purpose: The aim of this study was to investigate the influence of preoperative dry eye disease (DED) and ascorbic acid (AA) status on the outcomes of kerato-lenticule extraction (KLEx) surgery. The postoperative outcomes included visual and refractive parameters.

Method: A prospective, non-randomized controlled trial was conducted, and 68 patients who received KLEx surgery were included. These patients were divided according to their DED status and AA level, and a total number of 33, 19, and 16 patients/eyes were classified into non-DED, DED-high AA, and DED-low AA groups, respectively. The uncorrected distance visual acuity (UDVA), cycloplegia refraction, and AA concentration were determined before and after surgery. The Kruskal-Wallis test was used to investigate the postoperative outcomes among the three groups.

Results: One week postoperatively, the three groups presented similar UDVA and refraction values (all P > 0.05). One month postoperatively, however, the DED-low AA group demonstrated worse UDVA than the non-DED and DED-high AA groups (P = 0.012). After the KLEx surgery, the DED-low AA group still had the lowest AA level compared with the other two groups (both P < 0.05), and the decrease in AA was the largest in the DED-low AA group compared with the other two groups (both P < 0.05). High myopia was related to worse postoperative UDVA in the DED-high AA group (P = 0.034), and high myopia and large optic zone (OZ) were significantly correlated with worse postoperative UDVA in the DED-low AA group (both P < 0.05).

Conclusion: The concurrent presence of DED and a low baseline AA status was correlated with worse postoperative UDVA in patients who received KLEx surgery. Additionally, this population had more risk factors for poor postoperative UDVA than the other populations.

## Introduction

Corneal refractive surgeries are performed for refractive error correction, and different types of corneal refractive surgeries have been developed [1]. Some of the most common corneal refractive surgeries involve photorefractive keratectomy, laser in situ keratomileusis, and kerato-lenticule extraction (KLEx) [2]. A previous study found that the number of individuals requesting KLEx surgery for refractive correction has increased [3]. After KLEx surgery, the visual and refractive conditions are good, and more than 80% of eyes achieved an uncorrected distance visual acuity (UDVA) of 20/20 and a spherical equivalent (SE) deviation lower than ±1.00 diopter (D) [4].

Although the overall results of KLEx surgery are acceptable, some postoperative complications may diminish these outcomes [5]. Late corneal healing could lead to ocular irritation and reduced visual acuity after KLEx surgery [6]. Additionally, a residual refractive error that requires enhancement may occur, although the incidence is low [7]. However, dry eye disease (DED) is by far the most frequent complication after corneal refractive surgeries, including KLEx surgery, and it can significantly affect visual recovery [8]. The risk of developing severe DED is lower after KLEx surgery than after other refractive surgeries; however, advanced DED can develop in some patients who receive KLEx surgery [9], and those with previous DED may deteriorate after corneal refractive surgeries [2]. Thus, the co-factor of DED, which can influence postoperative visual acuity, should be examined.

Oxidative stress is a basic mechanism involved in multiple systemic and ocular diseases [10,11]. Increased oxidative stress has been found to be an indicator of DED and ocular surface damage [12], and corneal damage can be managed by utilizing antioxidants, including ascorbic acid (AA) [13,14]. However, the correlation between preoperative antioxidant concentrations and KLEx surgery outcomes has not been verified. Moreover, as oxidative stress and DED can damage the corneal surface [9,12], preoperative antioxidant levels and DED status may affect the outcomes of KLEx surgery.

Therefore, the objective of our study was to analyze the effect of DED and AA levels on the postoperative visual and refractive outcomes of KLEx surgery. The factors for worse postoperative conditions in individuals with different conditions were also compared.

## Materials and methods

Ethics statement

Our study complied with the 1964 Declaration of Helsinki and subsequent amendments. Our study was also approved by the Institutional Review Board of Linkou Chang Gung Memorial Hospital (project code: 202200858B0A3). Written informed consent was obtained from all participants, and our project was registered as a clinical trial at ClinicalTrials.gov (registration number: NCT05905237).

Participant selection

A prospective, non-randomized study was conducted at the Nobel Eye Institute, an ophthalmological institution specializing in cataract and keratorefractive surgeries. For inclusion in our study, the participants needed to satisfy the following inclusion criteria: (1) aged from 20 to 50 years; (2) myopia from -1.00 D to -10.00 D; (3) received KLEx surgery in any branch of the Nobel Eye Institute; and (4) realized the whole study project and signed the informed consent form. The following exclusion criteria were also established to enhance the homogeneity of the study participants: (1) UDVA worse than hand motion; (2) any cataract formation; (3) corneal diseases, including keratoconus, central corneal scars, and microbial keratitis and other corneal ectatic diseases; (4) retinal diseases, such as retinal detachment or macular pucker; (5) other prominent ocular diseases, such as end-stage glaucoma, uveitis, optic neuritis, or previous eyeball rupture; (6) an unstable refractive status that changed by more than 0.5 D in the past year; (7) pregnancy; and (8) active systemic inflammatory diseases, such as rheumatic arthritis or ankylosing spondylitis. Only the right eye of each person was included in our study. After selection, a total of 68 eyes were included in our study. These eyes were divided into those with DED and those without DED, according to the results of a preoperative Schirmer test, and those with DED were further separated into two groups based on the mean preoperative AA value. The systemic diseases we mentioned are hypertension, diabetes mellitus, asthma, and heart disease, and the ocular disease we mentioned is retinal degeneration. Finally, a total number of 33, 19, and 16 eyes were classified into a non-DED group, a DED-high AA group, and a DED-low AA group, respectively.

Surgical details

In our study, all KLEx surgeries were completed by one experienced refractive specialist (C.-K.C.). One femtosecond laser lenticule extraction device (VisuMax 500, Carl Zeiss, Jena, Germany) was used for the KLEx surgery. The optic zone (OZ) was set from 5.5 to 6.9 mm based on the pupil size and laser ablation depth, and a 3.0 mm corneal incision was made at 105°. After confirming the angle kappa via the coaxially sighted corneal light reflex maneuver and topography, the cornea was attached to the suction ring of the VisuMax 500. Subsequently, the VisuMax 500 struck the femtosecond laser, which created a corneal lenticule at approximately 23-25 seconds. After the complement of femtosecond laser emission, a specialized spatula was used to dissect the corneal lenticule, and then the corneal lenticule was extracted using another set of forceps.

Ophthalmic exam

All patients underwent the same preoperative examinations at the Nobel Eye Institute, which involved the following: UDVA, corrected distance visual acuity (CDVA), cycloplegic refraction of the sphere/cylinder power with an autorefractor (KR-8900, Topcon, Itabashi-ku, Tokyo, Japan), intraocular pressure (IOP) via pneumatic tonometry (NT-530, NIDEK, Gamagori, Aichi, Japan), keratometry (K), corneal astigmatism, angle kappa and central corneal thickness (CCT) via a tomographic device (Oculus Pentacam, OCULUS Optikgeräte GmbH, Münchholzhäuser, Wetzlar, Germany), and the Schirmer I test. For details of the Schirmer I test, a Schirmer strip was placed at the lateral 1/3 site of the inferior eyelid after topical anesthesia, and the patient closed their eyes for five minutes. The length of the wet part of the Schirmer strip was written down after removing the Schirmer strip. At our institution, the exams conducted after KLEx surgery include the UDVA, sphere power, cylinder power, and IOP, using the same device as used in the preoperative exam. The medical data obtained preoperatively, one week postoperatively, and one month postoperatively were analyzed.

Antioxidant determination

AA levels were determined using the same methods used in previous publications [14-16]. The tear film of the participant was taken near the incision site of the KLEx surgery, that is, the superior temporal site approximately 11 clock hours from the right eye. The Schirmer strip was placed near the limbal region of the corneal incision of the KLEx surgery for five minutes, and the tear film sample in the Schirmer strip was then placed in liquid nitrogen (-196°C) and stored in a refrigerator at approximately -80°C. The AA concentration determination was completed using a colorimetric OxiSelect™ Ascorbic Acid Assay Kit (FRASC, Cell Biolabs, Inc., San Diego, CA, USA), which confirmed the reduction of ferric ions by the function of ascorbic oxidase. Before analysis, all tear film samples preserved in the refrigerator at -80°C were thawed to nearly 4°C, and 35 μL of every tear film sample was diluted with an assay buffer of OxiSelect™ to 1/20 of the concentration. After this process, colorimetric assessments were performed within the light distances at a 540-600 nm wavelength using an absorbance microplate reader (Sunrise™ Tecan, Zurich, Switzerland). In the final step, the AA levels were confirmed using a colorimetric OxiSelect™ Ascorbic Acid Assay Kit. Regarding the antioxidant units, the AA concentrations were delineated in units of millimoles per liter (μmol/L or μM). To better ensure the precision of all tear film samples, each one was tested three times, and the average value of the three examinations was applied in the consecutive analyses.

Statistical analysis

IBM SPSS Statistics for Windows, Version 20 (Released 2011; IBM Corp., Armonk, New York) was used for all analyses conducted in our study. The Shapiro-Wilk test was used to verify the normal distribution of the whole KLEx group, and a non-normal distribution was found for all data (all P < 0.05). The statistical power of the current study was 0.75, with a 0.05 alpha value and a medium effect size, which was determined using G*Power software version 3.1.9.2 (Heinrich Heine University Düsseldorf, Düsseldorf, Germany). A descriptive analysis was used to present the initial data of the non-DED, DED-high AA, and DED-low AA groups. Then, the Kruskal-Wallis and Fisher's exact tests were used to compare the initial data values among the three groups according to each data type. The Wilcoxon signed-rank test was used to compare the preoperative and postoperative UDVA in different groups. Then, the Kruskal-Wallis test was also used to investigate the postoperative data and the AA level before and after the KLEx surgery among the three groups.

The trends in AA changes after the KLEx surgery among the three groups were investigated using the generalized linear mixed model with adjustments for age, sex, and preoperative cycloplegia refraction. The adjusted odds ratio (aOR) with the related 95% confidence interval (CI) of the AA changes among the three groups was produced. In the next steps, the generalized linear mixed model was used again to evaluate the association between preoperative factors and UDVA one month after the KLEx surgery, in which age and sex were adjusted. The preoperative factors investigated in our study included high myopia (more than -6.00 D of SE), high astigmatism (more than -3.00 D of cylinder power), steep K (average K more than 45 D), and large OZ (more than 6.5 mm). A P-value lower than 0.05 indicates statistical significance, and a P-value lower than 0.001 is presented as P < 0.001.

## Results

The initial characteristics of the three groups are displayed in Table 1. The mean age was 35.28±9.34, 36.51±8.64, and 35.97±9.55 years in the non-DED, DED-high AA, and DED-low AA groups, respectively. The mean age among the three groups did not reveal a significant difference (P = 0.725). Regarding the other demographic data, the sex distribution, rate of systemic disease, and presence of ocular disease did not show a significant difference among the three groups (all P > 0.05). The non-DED group exhibited a significantly higher Schirmer test value than the DED-high AA and DED-low AA groups (P < 0.001), whereas the other preoperative parameters demonstrated similar values among the three groups (all P > 0.05) (Table 1).

**Table 1:** The baseline characters of the study population * P < 0.05 denotes a significant difference among groups. The Kruskal-Wallis test and Fisher's exact test were used for the analysis. AA: ascorbic acid, CCT: central corneal thickness, CDVA: corrected distance visual acuity, D: diopter, DED: dry eye disease, IOP: intraocular pressure, LogMAR: logarithm of the minimum angle of resolution, OZ: optic zone, SD: standard deviation, SE: spherical equivalent.

Parameter	Non-DED Group	DED-High AA Group	DED-Low AA Group	P-value
Number of eyes	33	19	16	-
Age (mean ± SD)	35.28±9.34	36.51±8.64	35.97±9.55	0.725
Sex (male:female)	15:18	8:11	9:7	0.101
Systemic disease				0.455
0	27	14	13	-
1	5	4	2	-
≥2	1	0	1	-
Ocular disease				0.836
0	29	16	14	-
1	4	2	2	-
CDVA (LogMAR)	0.01±0.02	0.01±0.03	0.00±0.02	0.921
IOP (mmHg)	14.57±2.68	15.24±3.99	15.67±3.51	0.525
Sphere power (D)	4.85±2.27	4.98±2.61	4.49±2.45	0.231
Cylinder power (D)	1.35±1.08	1.20±1.17	1.36±1.13	0.167
SE (D)	5.53±2.36	5.58±2.54	5.17±2.50	0.258
K (D)	42.86±2.33	43.15±2.17	42.69±2.43	0.449
Angle kappa	0.16±0.08	0.15±0.10	0.16±0.11	0.963
Schirmer test (mm)	14.08±2.11	7.24±1.64	7.13±1.55	<0.001*
CCT (μm)	546.87±25.74	541.39±24.69	548.17±25.14	0.817
OZ (mm)	6.46±0.19	6.42±0.22	6.45±0.23	0.914

The mean preoperative UDVA was 0.85±0.38, 0.92±0.34, and 0.82±0.33 in the non-DED, DED-high AA, and DED-low AA groups, respectively, without significant differences (P = 0.530). One week postoperatively, the UDVA was 0.10±0.17, 0.09±0.14, and 0.18±0.15 in the non-DED, DED-high AA, and DED-low AA groups, respectively, with a marginal but not significant difference (P = 0.057). The sphere power, cylinder power, and IOP presented similar values between the three groups (all P > 0.05). One month postoperatively, however, the DED-low AA group demonstrated worse UDVA than the non-DED and DED-high AA groups (P = 0.012), whereas the other ophthalmic parameters showed insignificant differences among the three groups (all P > 0.05) (Table 2). All the non-DED (0.81), DED-high AA (0.87), and DED-low AA (0.72) groups showed significant improvements in mean UDVAs compared to preoperative statuses (all P < 0.001). The preoperative AA values are presented in Table 3, which shows that the DED-low AA group had the lowest AA value. After the KLEx surgery, the DED-low AA group still had the lowest AA value compared with the other two groups (both P < 0.05) (Table 3). Furthermore, the AA decrease was the largest in the DED-low AA group compared to the other two groups (both P < 0.05).

**Table 2:** The postoperative outcome after the refractive surgery * P < 0.05 denotes a significant difference among groups. The Kruskal-Wallis test was used for the analysis. AA: ascorbic acid, D: diopter, DED: dry eye disease, IOP: intraocular pressure, LogMAR: logarithm of the minimum angle of resolution, N: number, UDVA: uncorrected distance visual acuity.

Parameter	Non-DED Group (N = 33)	DED-High AA Group (N = 19)	DED-Low AA Group (N = 16)	P-value
Postoperative 1 week				
UDVA (LogMAR)	0.10±0.17	0.09±0.14	0.18±0.15	0.057
Sphere power (D)	0.27±0.22	0.20±0.24	0.19±0.25	0.628
Cylinder power (D)	0.16±0.15	0.18±0.14	0.16±0.17	0.789
IOP (mmHg)	13.59±2.35	13.21±2.25	13.76±2.42	0.341
Postoperative 1 month				
UDVA (LogMAR)	0.04±0.02	0.05±0.03	0.10±0.09	0.012*
Sphere power (D)	0.20±0.16	0.21±0.14	0.20±0.17	0.926
Cylinder power (D)	0.15±0.07	0.12±0.05	0.16±0.10	0.933
IOP (mmHg)	13.85±2.44	13.68±2.41	14.55±2.63	0.212

**Table 3:** The level of ascorbic acid before and after surgery between groups * P < 0.05 denotes a significant difference among groups. The Kruskal-Wallis test was used for the analysis. AA: ascorbic acid, DED: dry eye disease, N: number.

Time Point	Non-DED Group (N = 33)	DED-High AA Group (N = 19)	DED-Low AA Group (N = 16)	P-value
Preoperative (μM)	434.25±121.55	492.16±24.13	367.58±26.89	0.010*
Postoperative 1 week (μM)	345.15±96.22	407.68±31.52	214.63±39.34	<0.001*
Postoperative 1 month (μM)	360.61±100.27	423.32±35.77	216.58±44.37	<0.001*

Regarding the association between the preoperative factors and worse postoperative UDVA in the different groups, no significant association was found in the non-DED group (all P > 0.05) (Table 4). High myopia was related to worse postoperative UDVA in the DED-high AA group (aOR: 1.365, 95% CI: 1.010-1.578, P = 0.034). However, high myopia and large OZ were significantly correlated with worse postoperative UDVA in the DED-low AA group (both P < 0.05) (Table 4).

**Table 4:** The correlation between different parameters and postoperative visual acuity * P < 0.05 denotes a significant difference, indicating a significant correlation between that factor and the postoperative visual acuity. The generalized linear mixed model was used for the analysis. AA: ascorbic acid, aOR: adjusted odds ratio, CI: confidence interval, DED: dry eye disease, K: keratometry, OZ: optic zone.

Group	aOR	95% CI	P-value
Non-DED group			
High myopia	1.245	0.867-1.446	0.545
High astigmatism	1.085	0.824-1.323	0.687
Steep K	0.943	0.897-1.127	0.611
Large OZ	1.056	0.916-1.178	0.720
DED-high AA group			
High myopia	1.365	1.010-1.578	0.034*
High astigmatism	1.124	0.952-1.483	0.132
Steep K	0.996	0.875-1.351	0.788
Large OZ	0.968	0.837-1.284	0.513
DED-low AA group			
High myopia	1.418	1.115-1.682	0.009*
High astigmatism	1.208	0.997-1.465	0.054
Steep K	0.977	0.849-1.259	0.836
Large OZ	1.254	1.007-1.543	0.041*

## Discussion

In summary, patients with the concurrent presence of DED and a low AA status demonstrated worse postoperative UDVA after KLEx surgery than those without DED and those with DED and a high AA status. Moreover, the AA concentration in the DED-low AA group showed a greater decrease than in the other two groups. In addition, high myopia and large OZ were related to worse one-month postoperative UDVA in the DED-low AA group.

Among the etiologies of ocular surface damage, DED may be the most prominent cause, accounting for a large proportion of cases [17]. DED is caused by the inflammatory response and the subsequent unstable tear film [18]. Inflammatory cytokines, including interleukins, interferons, and tumor necrosis factor-alpha, are significantly elevated on the ocular surface of patients diagnosed with DED [19]. In addition, meibomian gland dysfunction, a crucial factor in the development of DED, is also an inflammatory disorder [20]. However, previous research has found that increased oxidative stress can also insult the ocular surface [12]. The presence of reactive oxygen species triggers the development of DED via the NLRP3-IL-1b signaling pathway [21]. Except for the above two conditions, undergoing corneal refractive surgery is another etiology of ocular surface injury [22]. The corneal incision and laser strike during corneal refractive surgery can lead to corneal nerve damage, which may influence the speed of corneal and visual recovery after the surgery [23].

AA is an antioxidant correlated to the formation of ocular disease in which the AA concentration in aqueous humor is significantly and negatively related to the severity of cataracts [16]. Moreover, the postoperative AA expressions in different types of refractive surgeries showed different values [15], and the application of AA can improve the corneal status in some ocular diseases, including Fuchs endothelial corneal dystrophy and pseudophakic bullous keratopathy via the reverse of oxidative stress-induced cell autophagy [14]. Because DED, corneal refractive surgery, and oxidative stress/low antioxidant conditions result in a worse ocular surface status [12,17,22], we speculate that the concurrent presence of DED and low antioxidant status may impact recovery after corneal refractive surgery. This concept is supported by the findings of our study.

The population with DED and a low baseline AA status presented worse UDVA after the KLEx surgery than the other two groups in our study. In the previous literature, both preoperative and postoperative DED were related to worse visual quality following corneal refractive surgeries [24]. Additionally, experimental research found that AA application contributed to faster corneal endothelial recovery [14]. However, whether the two factors have a dual effect on the outcomes of KLEx surgery has not yet been thoroughly evaluated. To the best of our knowledge, our findings may be a preliminary indication of the correlation between DED and low AA conditions with worse outcomes after KLEx surgery. In addition, the baseline ophthalmic indices were largely similar among the three groups, except for the Schirmer test results, which were applied for the grouping; thus, the homogeneity of the three groups should be acceptable.

Furthermore, the AA determination and analysis among the three groups were conducted simultaneously, and the surgical time among the three groups was also randomly scheduled. As a consequence, the DED plus low preoperative AA concentrations may be independently associated with worse postoperative UDVA after KLEx surgery. The persistent ocular surface damage, elevated oxidative stress, and coexisting DED could affect visual recovery after corneal refractive surgeries [25]. Furthermore, according to a previous study, inflammation in the anterior segment of the eye is correlated with higher degrees of oxidative stress [26]. As a result, the concurrent presence of DED and a low AA status may contribute to prominently higher postoperative inflammation and oxidative stress, thus reducing the recovery of corneal tissue and visual acuity. In our study, refraction was not influenced by either the DED status or AA level, which may indicate that the predictability of KLEx surgery is still acceptable, even under slower visual recovery, and that the rate of enhancement might not be influenced by such conditions.

Regarding the degree of AA changes among the different groups, all the populations with different baseline AA expressions demonstrated a prominent decrease in AA after the KLEx surgery. Regarding oxidative stress after corneal refractive surgery, previous studies revealed elevated oxidative stress after the femtosecond laser-based flap creation [27]. Because the creation of a corneal lenticule in KLEx surgery resembles the flap creation step in laser in situ keratomileusis [3], it is possible that antioxidants, including AA, were reduced after the KLEx surgery due to laser-related oxidative stress.

Regarding the changes in AA in the different groups, the group with a low AA status demonstrated the largest decrease in AA after the KLEx surgery. Higher oxidative stress was observed in the population with DED, contributing to the tear film deficiency and the antioxidant decrease in the tear film [28]; thus, oxidative stress may more easily increase in those with DED after corneal refractive surgery. Furthermore, postoperative DED after refractive surgery is usually accompanied by increased inflammation, which may further consume the antioxidants on the ocular surface [29]. As a consequence, we speculate that the inadequate baseline antioxidant levels in the DED-low AA group may not compensate for the inflammation and oxidative stress after KLEx surgery, and the residual postoperative oxidative stress and pre-existing DED further contribute to antioxidant consumption.

In the DED-high AA group, although the influence of DED on oxidative stress elevation could persist, the high baseline AA concentration may compensate for the DED-related oxidative stress and prevent subsequent reactive oxygen species formation after the KLEx surgery. As the change in AA may be related to visual performance in our study, management to prevent AA reduction should be evaluated.

Regarding the correlation between the preoperative parameters and postoperative UDVA in the different groups, a high-myopia status correlated with worse postoperative UDVA in both the DED-high AA and DED-low AA groups. This is the first study to display this phenomenon. High myopia is related to an inflammatory condition of the eye [30]. Because inflammation can induce oxidative stress [29], high-myopia patients may experience a significant increase in oxidative stress after undergoing corneal refractive surgery, which may affect corneal healing and consecutive visual recovery. However, in the previous literature, visual recovery after corneal refractive surgery in a high-myopia population was significantly slower than that in a low-myopia population; thus, the low antioxidant status and DED in such conditions may further affect postoperative UDVA. In addition to the degree of myopia, a large OZ was also associated with worse postoperative UDVA in the DED-low AA group but not in the other two groups. A large OZ in corneal refractive surgery indicates a higher laser intensity during the surgery [3]. Although the laser amount in KLEx surgery is not as much related to the treatment area as in laser in situ keratomileusis [5], a higher laser amount is still applied for a large OZ. The higher laser amount applied to those with DED and AA may influence postoperative visual recovery.

There are certain limitations to our study. Firstly, we did not randomize the participants into different groups, which could have reduced the homogeneity of the study population and influenced the subsequent results. Furthermore, the number of eyes in our study was too low, with only 68 eyes included in the analysis, which could have led to statistical bias. In addition, we only determined the presence of DED according to the results of the Schirmer test and without a formal DED evaluation; thus, the DED type was not investigated. Besides, the social factors and diet factors were not accounted for in our study. Finally, we measured the AA expressions until one month postoperatively, which is a relatively inadequate follow-up period for recovery after KLEx surgery.

## Conclusions

In conclusion, the concurrent presence of DED and a low baseline AA status correlated with worse UDVA after KLEx surgery. Furthermore, postoperative UDVA was affected by more preoperative parameters in those with DED and a low baseline AA status. Consequently, an antioxidant supplement may be considered for those with DED and a low baseline AA and scheduled for KLEx surgery. Further large-scale prospective studies are required to investigate the effect of preoperative antioxidant supplements on visual recovery after KLEx surgery.
